# Dietary patterns and depressive symptoms in a UK cohort of men and women: a longitudinal study

**DOI:** 10.1017/S1368980017002324

**Published:** 2017-09-18

**Authors:** Kate Northstone, Carol Joinson, Pauline Emmett

**Affiliations:** 1 The National Institute for Health Research Collaboration for Leadership in Applied Health Research and Care West (NIHR CLAHRC West), University Hospitals Bristol NHS Foundation Trust, Bristol, UK; 2 School of Social and Community Medicine, NIHR CLAHRC West, Level 9, Whitefriars, Lewins Mead, University of Bristol, Bristol BS1 2NT, UK

**Keywords:** Depression, ALSPAC, Dietary patterns, Principal components analysis, Edinburgh Postnatal Depression Scale

## Abstract

**Objective:**

There is evidence to suggest that individual components of dietary intake are associated with depressive symptoms. Studying the whole diet, through dietary patterns, has become popular as a way of overcoming intercorrelations between individual dietary components; however, there are conflicting results regarding associations between dietary patterns and depressive symptoms. We examined the associations between dietary patterns extracted using principal component analysis and depressive symptoms, taking account of potential temporal relationships.

**Design:**

Depressive symptoms in parents were assessed using the Edinburgh Postnatal Depression Scale (EPDS) when the study child was 3 and 5 years of age. Scores >12 were considered indicative of the presence of clinical depressive symptoms. Diet was assessed via FFQ when the study child was 4 years of age.

**Setting:**

Longitudinal population-based birth cohort.

**Subjects:**

Mothers and fathers taking part in the Avon Longitudinal Study of Parents and Children when their study child was 3–5 years old.

**Results:**

Unadjusted results suggested that increased scores on the ‘processed’ and ‘vegetarian’ patterns in women and the ‘semi-vegetarian’ pattern in men were associated with having EPDS scores ≥13. However, after adjustment for confounders all results were attenuated. This was the case for all those with available data and when considering a sub-sample who were ‘disease free’ at baseline.

**Conclusions:**

We found no association between dietary patterns and depressive symptoms after taking account of potential confounding factors and the potential temporal relationship between them. This suggests that previous studies reporting positive associations may have suffered from reverse causality and/or residual confounding.

Depression is one of the leading causes of disease burden across the world^(^ ^1^ ^)^. In the UK, there is evidence to suggest that almost a fifth of the adult population suffers from depression/anxiety^(^ ^2^ ^)^ with a peak prevalence between 45 and 54 years of age. Women are more than twice as likely as men to be treated for depression^(^ ^3^ ^)^.

Dietary intake is a modifiable risk factor for many diseases and has been implicated as a risk factor for depression. However, the role of nutrition in the development of depression is not clear. A number of studies have reported associations between individual nutrient and food intakes and the prevalence of depression^(^ ^4^ ^)^, with protective effects being shown with fish and *n*-3 fatty acids, fruit and vegetables^(^ ^5^ ^)^. However, studying foods or nutrients alone can be problematic due to the intercorrelations between them and their potentially interactive effects^(^ ^6^ ^)^. The use of dietary patterns enables study of the diet as a whole by reducing a large number of food intake variables into a handful of variables which best describe the overall dietary types in a population. A popular method of deriving such patterns is through principal components analysis (PCA). This method reduces a large number of correlated food intake variables into a small number of uncorrelated factors, thus summarising the complex data while maintaining the dimensionality of the diet.

A number of studies have examined cross-sectional associations between dietary patterns obtained using PCA and depression in diverse populations (see Rahe *et al.* for a review^(^ ^7^ ^)^) and suggest that a ‘healthy’ or ‘traditional’ pattern may have a protective effect on depression. However, these studies are limited by their cross-sectional nature and the alternative explanation of reverse causality cannot be excluded. In other words, it is quite possible that the presence of depression in an individual may cause them to alter the way in which they eat; possibly to enhance mood or because when depressed, individuals may turn to ‘more convenient’ foods^(^ ^8^ ^)^. Alternatively, a depressed individual’s appetite or food preferences could be altered^(^ ^9^ ^)^.

Recently, a flurry of prospective studies have been published^(^ ^10^ ^–^ ^13^ ^)^ with conflicting results. However, two of these focused on pregnant women^(^ ^10^ ^,^ ^13^ ^)^, a time when diet may differ from habitual intake, or were performed in women only^(^ ^11^ ^)^, and none have been performed in the UK.

The aim of the current study was to examine the possible effects of dietary patterns on depressive symptoms in a population-based UK cohort of men and women. To overcome the key issue of reverse causality, we repeated the analysis with a ‘disease free’ group by selecting those who did not have excessive depressive symptoms at baseline. We examined the effect of dietary patterns obtained one year later on depressive symptoms assessed a subsequent year after that.

## Methods

The Avon Longitudinal Study of Parents and Children (ALSPAC) is an ongoing population-based study designed to investigate environmental, genetic and other effects on the health and well-being of children^(^ ^14^ ^)^. Pregnant women living in the former Avon Health Authority in South West England with an expected date of delivery between 1 April 1991 and 31 December 1992 were eligible to enrol, resulting in a cohort of 14 541 pregnancies. Partners of the women were invited to partake in the study by the women; they were not contacted directly by the study team. The primary source of data collection for both the woman and her partner was via self-completion questionnaires. The study website contains details of all data that are available through a fully searchable data dictionary (http://www.bris.ac.uk/alspac/researchers/data-access/data-dictionary/). Ethical approval for the study was obtained from the ALSPAC Ethics and Law Committee and the Local Research Ethics Committees.

When the study child in the family was 4 years of age, a questionnaire was sent to the woman and a separate one was sent for her to pass on to her partner if she chose to. Both contained an FFQ: a set of questions enquiring about the frequency of consumption of a wide variety of foods and drinks. This FFQ was modified slightly from a version first used in the mothers during pregnancy^(^ ^15^ ^)^. This FFQ has been shown to produce mean nutrient intakes^(^ ^15^ ^)^ similar to those obtained for women in the British National Diet and Nutrition Survey for adults^(^ ^16^ ^)^. The participants were asked to indicate how often they were currently consuming each food type using the following options: (i) ‘never or rarely’; (ii) ‘once in 2 weeks’; (iii) ‘1–3 times a week’; (iv) ‘4–7 times a week’; and (v) ‘more than once a day’. Responders were asked to report the number of cups of tea or coffee (but not serving size) and slices of bread consumed daily, together with the type of bread and milk usually consumed. The response options data were numerically transformed into times per week as follows: (i) 0; (ii) 0·5; (iii) 2; (iv) 5·5; and (v) 10. Since some variables (e.g. bread) were measured on a different scale (such as daily rather than weekly), all frequency variables were standardized by subtracting the mean and dividing by the sd for each variable. Dietary patterns were extracted from the women and the men separately using PCA as described below.

When the study child was almost 3 years of age, both mothers and partners completed questionnaires which contained the Edinburgh Postnatal Depression Scale (EPDS)^(^ ^17^ ^)^. This was repeated when the study child was 5 years of age. Although the EPDS was developed to screen for depression in women postnatally, it has been found to be useful in women outside the postnatal period and also in men^(^ ^18^ ^–^ ^20^ ^)^. The EPDS measures depressive symptoms, but it has been shown that a score of >12 is strongly predictive in women of clinical depression^(^ ^17^ ^)^ and has been used to indicate probable depressive disorder^(^ ^21^ ^)^. EPDS scores >12 have a high specificity and sensitivity in predicting clinically diagnosed depressive disorder. Scoring >12 on the EPDS on at least two occasions suggests depression that is likely to require treatment^(^ ^22^ ^)^. The EPDS comprises ten items inquiring about the frequency of experiencing depressive symptoms in the past week. Each item has four response categories scored from 0 to 3, and scores on the scale range from 0 to 30. It is the only self-report scale that has been validated for use in the antenatal period^(^ ^20^ ^)^.

A number of social and demographic factors were investigated as potentially explaining any evident associations between dietary pattern and depressive symptoms. Age was derived for women as that at delivery of the study child; for men, this was the self-reported age at which they completed the 3-year questionnaire. Highest education and ethnicity of both parents were reported by the mother during pregnancy. Women and their partners provided data on a range of confounders at 3 years (see Table 1). These included housing tenure, marital status and subjective health status (measured as the response to the question ‘Which of the following would you say describes your health now?’: ‘fit and well’; ‘mostly well and healthy’; ‘often feel unwell’; ‘hardly ever feel well’. The last two categories were combined for the present analysis as very few reported hardly ever feeling well). Overcrowding was defined as there being more than one person per room in the household excluding kitchen and bathroom. Self-reported symptoms of anxiety were derived from scores from the anxiety subscale of the Crown Crisp Experiential Index (CCEI)^(^ ^23^ ^)^. The anxiety scale of the CCEI comprises eight questions inquiring about symptoms of free-floating anxiety. Respondents were asked to indicate the current frequency of experiencing these symptoms (‘very often’, ‘often’, ‘not very often’, ‘never’) and scores on the scale range from 0 to 16 (internal consistency for the anxiety scale exceeded 0·80 in this sample).

**Table 1:** Characteristics of the whole cohort (i.e. unrestricted sample) of women and men from the Avon Longitudinal Study of Parents and Children

	Women (*n* 7698)		Men (*n* 3082)	
	*n*	%	*n*	%
Age (years)				
≤29	4298	55·8	506	16·4
30–34	2501	32·5	1188	38·5
35–39	791	10·3	871	28·3
≥40	108	1·4	517	16·8
Highest education				
<O level*	1714	22·7	571	19·0
O level	2685	35·6	647	21·5
≥O level	3149	41·7	1790	59·5
Ethnicity				
White	7417	98·5	2989	98·6
Non-white	113	1·5	43	1·4
Housing tenure				
Owner-occupied	6335	82·5	2720	89·1
Council/housing association	884	11·5	172	5·6
Private rented/other	456	5·9	160	5·2
Marital status				
Married	6323	82·7	2841	92·6
Single/widowed/divorced	1319	17·3	228	7·4
Subjective health status				
Fit and well	3834	50·9	1939	64·4
Mostly well	3314	44·0	993	33·0
Often unwell	390	5·1	80	2·6
Overcrowded accommodation				
Yes†	7014	93·2	2841	94·8
No	512	6·8	155	5·2
Anxiety score (CCEI)				
Mean and sd	4·62	3·50	2·93	2·66
Median and IQR	4	2–6	2	1–4

CCEI, Crown Crisp Experiential Index; IQR, interquartile range.

* O levels were the academic examinations taken at 16 years of age in the UK school system.

† More than one person per room in the household excluding kitchen and bathroom.

The questionnaire to the partners asked whether the person completing it was the natural father of the child. All analyses are restricted to those with a positive response to this question; we chose this restriction primarily to ensure that any possible female partners were excluded (women were asked to pass questionnaires to their current partner regardless of their gender) as we wanted to ensure the male sample only included males.

### Statistical methods

The dietary patterns of the women and the men were obtained independently using PCA with varimax rotation^(^ ^24^ ^)^. The methodology has been reported elsewhere^(^ ^25^ ^,^ ^26^ ^)^. Briefly, the number of components that best represented the data was chosen based on the scree plot^(^ ^27^ ^)^ together with the interpretability of the factor loadings. Individuals were excluded from the PCA if more than ten dietary items had missing data (*n* 17 and *n* 50 for women and men, respectively); for those with ten or fewer missing items, it was assumed that the missing items were not consumed and were given the value of 0. Component scores were calculated by multiplying the factor loadings for each component by the corresponding standardised value for each food and summing across the food items. Items with loadings above |0·3| on a component were considered the most informative in describing the patterns. Labels were assigned to the extracted dietary patterns according to the loadings.

EPDS scores were considered as a binary variable (>12 *v*. ≤12)^(^ ^17^ ^)^, given the skewed nature of the continuous variable and the fact that the score is used as a screening tool with this cut-off. Logistic regression analyses were performed to obtain odds ratios and 95 % confidence intervals. Due to the different patterns obtained and the higher prevalence of depressive symptoms in women, the analyses were performed for men and women separately. We repeated our analyses in those who were not defined as having high levels of depressive symptoms at baseline (3 years) to achieve a ‘disease free’ sample. Women and men who scored ≥13 at baseline were excluded to create a restricted sample to better examine the temporal relationship between diet and depressive symptoms.

## Results

**Fig. 1:**
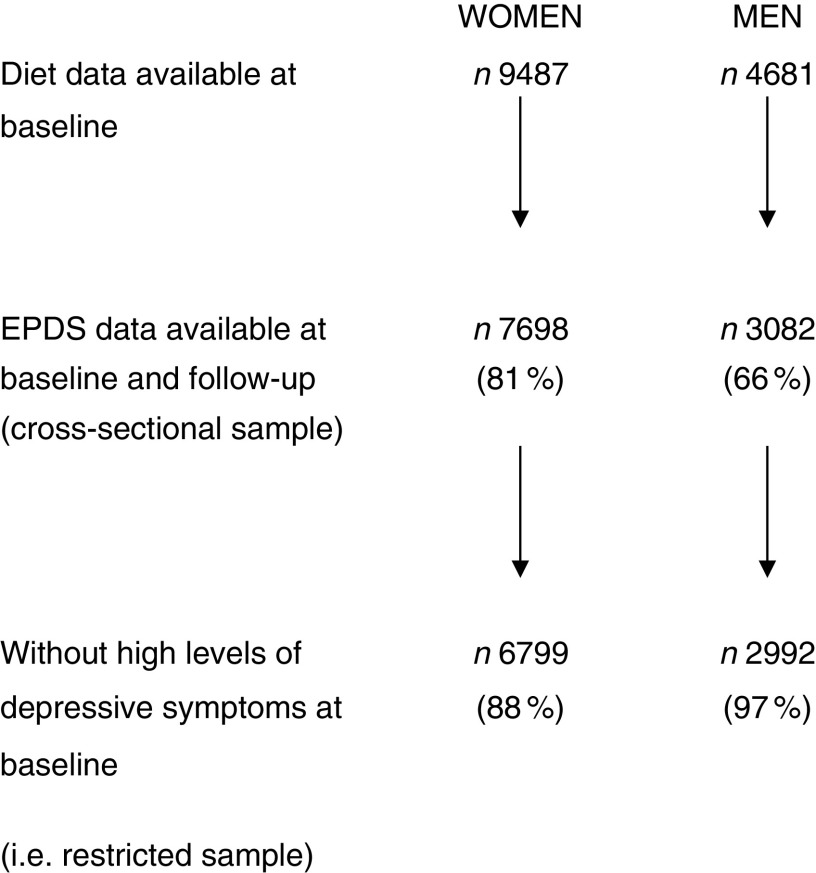
Women and men from the Avon Longitudinal Study of Parents and Children included in the present study on dietary patterns and depressive symptoms (EPDS, Edinburgh Postnatal Depression Scale)

As shown in Fig. 1, 9487 women and 4681 men had complete FFQ data at 4 years. Of these, EPDS data were available both at baseline and follow-up for 7698 (81 %) women and 3082 (66 %) men. Of those with complete data, mean EPDS (sd) scores at 5 years were 5·94 (4·98) and 3·81 (3·92) for women and men, respectively. Median (interquartile range) EPDS scores were 4 (2–6) for women and 2 (1–4) for men. Of women, 899 (11·7 %) were classified as having high levels of depressive symptoms at baseline (according to an EPDS score ≥13) and were excluded from the longitudinal analysis, compared with ninety (2·9 %) men who were initially above the cut-off and therefore excluded. Differences between participants with EPDS scores and those without are shown in the online supplementary material, Supplemental Table 1. Those excluded from the analysis were more likely to be younger, lower educated and live in overcrowded conditions. They were less likely to live in owner-occupied accommodation or to be married.

Four patterns were obtained in men^(^ ^25^ ^)^ and in women^(^ ^26^ ^)^ with some similarities. The first dietary pattern was labelled ‘health conscious’ in both and was associated with brown/wholemeal bread, pasta, rice, salad and fruit. In women, additional foods loading highly on this pattern included all vegetables, pulses and fish. In the men, the second component had high loadings for all types of vegetables, potatoes, red meat and poultry, and was therefore labelled ‘traditional’ as it represents the traditional British ‘meat and two veg’ diet. This component was not extracted in the women. The third component in the men was described by high loadings for processed and high-fat foods such as sausages/burgers, coated poultry products, pizza, chips and crisps (potato chips). There were also high loadings for high-sugar products such as biscuits, sweets, chocolates, puddings, fizzy drinks and squash. This pattern was therefore labelled ‘processed/confectionery’ as it was a combination of the ‘processed’ and ‘confectionery’ patterns that were obtained as the second and third components in the women. The final pattern extracted for the women was labelled the ‘vegetarian’ pattern as it was characterised by high intakes of meat substitutes, pulses, nuts and fish and it was also negatively associated with meats. A similar pattern was obtained in the men but the loadings for poultry and cold meats were not highly negative, and we therefore labelled this pattern ‘semi-vegetarian’.

We have previously reported the associations between dietary pattern scores and sociodemographic characteristics^(^ ^25^ ^,^ ^26^ ^)^. In brief, increasing scores on the ‘health-conscious’ components were associated with higher educational attainment, being white, owner-occupied housing and older age, whereas the ‘processed’ and ‘processed/confectionery’ patterns showed associations that were the reverse of these.

Table 2 presents the results of the logistic regression analyses examining the odds of having high levels of depressive symptoms according to a 1 sd increase in dietary pattern score in the women. There was an unadjusted association between the ‘processed’ pattern and depressive symptoms (OR=1·12; 95 % CI 1·04, 1·20). However, this was lost after adjustment for other variables (adjusted OR=1·01; 95 % CI 0·92, 1·10). Restricting the sample to those who were ‘disease free’ at baseline reduced the unadjusted OR slightly to 1·09 (95 % CI 1·00, 1·19), which again was lost after adjustment.

**Table 2:** Associations between continuous dietary pattern scores and binary EPDS scores* in the whole cohort (i.e. unrestricted sample) and the restricted sample (who were ‘disease free’ at baseline) of women from the Avon Longitudinal Study of Parents and Children

	Whole cohort			Restricted sample		
Women’s dietary pattern	OR	95 % CI	*P* value	OR	95 % CI	*P* value
Unadjusted	*n* 7698			*n* 6799		
‘Health conscious’	0·94	0·87, 1·01	0·082	0·99	0·90, 1·09	0·830
‘Processed’	1·12	1·04, 1·20	0·002	1·09	1·00, 1·19	0·064
‘Confectionery’	0·99	0·92, 1·06	0·754	1·04	0·95, 1·13	0·445
‘Vegetarian’	1·06	0·99, 1·13	0·120	1·05	0·96, 1·15	0·296
Adjusted†	*n* 7134			*n* 6318		
‘Health conscious’	0·94	0·87, 1·03	0·180	1·01	0·91, 1·11	0·913
‘Processed’	1·01	0·92, 1·10	0·855	1·04	0·94, 1·16	0·420
‘Confectionery’	1·01	0·93, 1·10	0·777	1·05	0·95, 1·15	0·335
‘Vegetarian’	0·94	0·87, 1·02	0·134	1·01	0·92, 1·11	0·814

EPDS, Edinburgh Postnatal Depression Scale.

* EPDS score ≥13 compared with the reference group having EPDS score <13.

† Adjusted for all variables in Table 1.

In the men (Table 3), there was an association between the ‘semi-vegetarian’ pattern and having high levels of depressive symptoms in the unadjusted analyses (OR=1·20; 95 % CI 1·04, 1·38). However, on adjustment, the effect was lost. Similar effect sizes were evident in both the cross-sectional and the ‘disease free’ samples before and after adjustment.

**Table 3:** Associations between continuous dietary pattern scores and binary EPDS scores* in the whole cohort (i.e. unrestricted sample) and the restricted sample (who were ‘disease free’ at baseline) of men from the Avon Longitudinal Study of Parents and Children

	Whole cohort			Restricted sample		
Men’s dietary pattern	OR	95 % CI	*P* value	OR	95 % CI	*P* value
Unadjusted	*n* 3080			*n* 2992		
‘Healthy’	0·90	0·75, 1·07	0·235	1·01	0·83, 1·24	0·921
‘Traditional’	0·92	0·77, 1·11	0·903	0·98	0·80, 1·21	0·861
‘Processed/confectionery’	0·95	0·78, 1·15	0·581	0·85	0·67, 1·06	0·142
‘Semi-vegetarian’	1·20	1·04, 1·38	0·011	1·21	1·05, 1·39	0·008
Adjusted†	*n* 2826			*n* 2746		
‘Healthy’	0·99	0·75, 1·29	0·925	1·08	0·80, 1·47	0·602
‘Traditional’	0·99	0·76, 1·30	0·951	1·09	0·81, 1·48	0·568
‘Processed/confectionery’	0·88	0·66, 1·17	0·380	0·80	0·68, 1·11	0·187
‘Semi-vegetarian’	0·86	0·64, 1·16	0·315	0·85	0·62, 1·16	0·303

EPDS, Edinburgh Postnatal Depression Scale.

* EPDS score ≥13 compared with the reference group having EPDS score <13.

† Adjusted for all variables in Table 1.

## Discussion

In this large cohort study, we have found little evidence to support the hypothesis that dietary patterns extracted using PCA are related to depressive symptoms in women or men. All associations were lost after several potential confounding factors were taken into account. This was true in both a cross-sectional analysis as well as when considering a sub-sample who were ‘disease free’ at baseline.

A number of cross-sectional studies have examined the association between dietary patterns and depression. One study in Japan found no associations^(^ ^13^ ^)^. However, another reported a protective effect of a ‘healthy’ dietary pattern characterised by high intakes of fruit, vegetables and soya products on depressive symptoms^(^ ^28^ ^)^. Similar associations were seen in an elderly French population^(^ ^29^ ^)^. Oddy *et al*.^(^ ^30^ ^)^ reported poorer mental health in Australian adolescents who scored higher on a ‘Western’ pattern (high in red/processed meats, confectionery and refined foods), while Jacka *et al*.^(^ ^12^ ^)^ reported increased odds of depression with their ‘Western’ pattern but reduced odds of depression with their ‘traditional’ diet. More recent studies that have taken any temporal relationship into account found mixed results. Jacka *et al*.^(^ ^12^ ^)^ reported in >60-year-old Australians that future depression was associated with increased baseline scores on a ‘Western’ pattern and lower baseline scores on a ‘Healthy’ pattern. Meanwhile, no associations were reported between dietary pattern scores and subsequent depression in Chinese older people^(^ ^9^ ^)^ or American middle-aged and older women^(^ ^11^ ^)^.

The above studies were either (i) cross-sectional, and therefore the researchers could not accurately determine the direction of causality, or (ii) longitudinal, and although those studies assessed diet before depression, reverse causality could still not be ruled out since previous depression was not accounted for. In the current study, we attempted to tackle this by restricting the sample to a group who were ‘disease free’ at baseline. In other words, we selected a group who were not classified as having high levels of depressive symptoms (according to EPDS score >12) and followed them up collecting dietary data a year after baseline and then reassessing their depressive symptoms a further year later. A potential limitation of this method is that we cannot be sure that our selected group had never had depressive symptoms prior to baseline nor can we be sure that an individual’s dietary pattern did not change in the time elapsing between dietary assessment and the second measure of depressive symptoms. Indeed, it could be argued that by restricting the sample we have excluded the long-term cases of depression who have potentially always consumed a poor diet. In addition, it should be acknowledged that depression is primarily a chronic condition and we may not expect too many incident cases to appear over the study period. Nevertheless, our results are similar in the two sets of analyses. Taken together with the fact that no associations were seen in our unrestricted sample after adjustment, this suggests that previous studies were likely to suffer from residual confounding rather than showing any true causal relationship. We did not choose to exclude individuals based on their own self-report of depression. Self-report is unlikely to be reliable and may introduce unnecessary bias, so it was felt that using the EPDS was methodologically more appropriate than relying on self-report.

Despite the results reported here examining overall diet, we cannot rule out the possibility that individual aspects of the diet may be associated with the development of depressive symptoms. For example, a recent meta-analysis reported that increased fish consumption was protective against depression in both men and women^(^ ^31^ ^)^. If there is a true biological mechanism for any particular aspect of the diet acting on mental well-being, this may be diluted or lost altogether when examining the diet as a whole. In addition, we did not investigate whether individuals consumed any nutritional supplements; the focus of the study was on examining the diet as a whole and therefore was based on food intake. It is also possible that using other methods of assessing the whole diet, such as cluster analysis or established predefined dietary indices, may give different results from those presented here.

The strengths of the current study include the large sample size and the ability to adjust for a wide range of confounding variables to minimise residual confounding. We have examined both continuous and binary outcomes and used a novel way of attempting to unravel any temporal relationship between exposure and outcome in this longitudinal cohort study. Nevertheless, there are several limitations that must be acknowledged. Limitations include the self-report of both dietary intake and depressive symptoms, both of which may be subject to bias. To minimise the latter, we used an established and well-validated measure in the form of the EPDS. The EPDS is a screening tool rather than a diagnostic tool^(^ ^17^ ^,^ ^18^ ^)^. A further limitation of the study with respect to men is that those who took part were, by design, invited to do so by their partners. It is possible that men living on their own may have (i) different diets compared with those cohabiting with partners and/or (ii) a higher prevalence of depressive symptoms. Therefore, our results cannot be generalized to all UK men.

## Conclusion

In conclusion, in this sample of UK adults, it is unlikely that overall dietary patterns contribute to the development of depressive symptoms. The findings reported here are based on a credible methodology taking account of both the temporal relationship between diet and depression and examining many potential confounders. We therefore suggest that residual confounding may explain the previous positive relationships that have been reported in the literature when examining the diet as a whole.

## Supplementary material

For supplementary material accompanying this paper visit https://doi.org/10.1017/S1368980017002324. click here to view supplementary material
