# Supplementary material for: Dietary patterns and depressive symptoms in a UK cohort of men and women: a longitudinal study
Source: Public Health Nutr. 2017 Sep 18;21(5):831–7. doi: 10.1017/S1368980017002324 (PMC5848748; doi:10.1017/S1368980017002324)
Supplement: Supplementary file 1 [file S1368980017002324sup001.docx]

**Supplemental** **Table 1: Characteristics of women and men in the sample with and without EPDS at baseline and follow-up (differences assessed using X^2^ test).**

|  | **Women** | | **Men** | |
| --- | --- | --- | --- | --- |
|  | **Without EPDS**  **n=1789** | **With EPDS**  **n=7698** | **Without EPDS**  **n=1599** | **With EPDS**  **n=3082** |
| **Age**  ≤ 29  30-34  35-39  40+ | 1213 (67.8%)  428 (23.9%)  131 (7.3%)  17 (1.0%) | 4298 (55.8%)  2501 (32.5%)  791 (10.3%)  108 (1.4%)  P<0.001 | 963 (60.2%)  311 (19.4%)  206 (12.9%)  119 (7.4%) | 506 (16.4%)  1188 (38.5%)  871 (28.3%)  517 (16.8%)  P<0.001 |
| **Highest Education**  < O level*  O level  ≥ O level | 607 (37.3%)  575 (35.3%)  446 (27.4%) | 1714 (22.7%)  2685 (35.6%)  3149 (41.7%)  P<0.001 | 423 (27.9%)  355 (23.4%)  737 (48.6%) | 571 (19.0%)  647 (21.5%)  1790 (59.5%)  P<0.001 |
| **Ethnicity**  White  Non-white | 1823 (98.2%)  34 (1.9%) | 7417 (98.5%)  113 (1.5%)  P=0.023 | 1511 (98.1%) 29 (1.9%) | 2989 (98.6%)  43 (1.4%)  P=0.148 |
| **Housing tenure**  Owner-occupied  Council/Housing assoc  Private rented/Other | 660 (69.5%)  195 (20.5%)  94 (9.9%) | 6335 (82.5%)  884 (11.5%)  456 (5.9%)  P<0.001 | 1149 (81.7%)  169 (12.0%)  88 (6.3%) | 2720 (89.1%)  172 (5.6%)  160 (5.2%)  P<0.001 |
| **Marital status**  Married  Single/widowed/divorced | 704 (74.8%)  237 (25.2%) | 6323 (82.7%)  1319 (17.3%)  P<0.001 | 742 (88.4%)  97 (11.6%) | 2841 (92.6%)  228 (7.4%)  P<0.001 |
| **Subjective health status**  Fit and well  Mostly well  Often unwell | 460 (50.4%)  393 (43.1%)  59 (6.5%) | 3834 (50.9%)  3314 (44.0%)  390 (5.1%)  P=0.254 | 501 (61.2%)  291 (35.5%)  27 (3.3%) | 1939 (64.4%)  993 (33.0%)  80 (2.6%)  0.197 |
| **Overcrowded accommodation**  Yes **  No | 827 (89.5%)  97 (10.5%) | 7014 (93.2%)  512 (6.8%)  P<0.001 | 1257 (90.7%)  129 (9.3%) | 2841 (94.8%)  155 (5.2%)  P<0.001 |
| **Anxiety score (CCEI)**  mean (SD)  median (IQR) | 5.01 (3.82%)  4 (1, 6) | 4.62 (3.50)  4 (2, 6) | 2.99 (2.74%)  2 (1, 4) | 2.93 (2.66)  2 (1, 4) |

*O levels were the academic examinations taken at 16 years of age in the UK school system

** More than 1 person per room in the household excluding kitchen and bathroom
